# Roles for the 3D genome in the cell cycle, DNA replication, and double strand break repair

**DOI:** 10.3389/fcell.2025.1548946

**Published:** 2025-02-27

**Authors:** Katherine A. Giles, Phillippa C. Taberlay, Anthony J. Cesare, Mathew J. K. Jones

**Affiliations:** ^1^ Children’s Medical Research Institute, University of Sydney, Westmead, NSW, Australia; ^2^ Menzies Institute for Medical Research, University of Tasmania, Hobart, TAS, Australia; ^3^ Faculty of Medicine, Frazer Institute, University of Queensland, Brisbane, QLD, Australia; ^4^ School of Chemistry and Molecular Biosciences, University of Queensland, Brisbane, QLD, Australia

**Keywords:** chromatin, replication, DNA repair, DNA damage, TADs, cell cycle, chromatin organisation, 3D genome architecture

## Abstract

Large eukaryotic genomes are packaged into the restricted area of the nucleus to protect the genetic code and provide a dedicated environment to read, copy and repair DNA. The physical organisation of the genome into chromatin loops and self-interacting domains provides the basic structural units of genome architecture. These structural arrangements are complex, multi-layered, and highly dynamic and influence how different regions of the genome interact. The role of chromatin structures during transcription via enhancer-promoter interactions is well established. Less understood is how nuclear architecture influences the plethora of chromatin transactions during DNA replication and repair. In this review, we discuss how genome architecture is regulated during the cell cycle to influence the positioning of replication origins and the coordination of DNA double strand break repair. The role of genome architecture in these cellular processes highlights its critical involvement in preserving genome integrity and cancer prevention.

## Introduction

**FIGURE 1:**
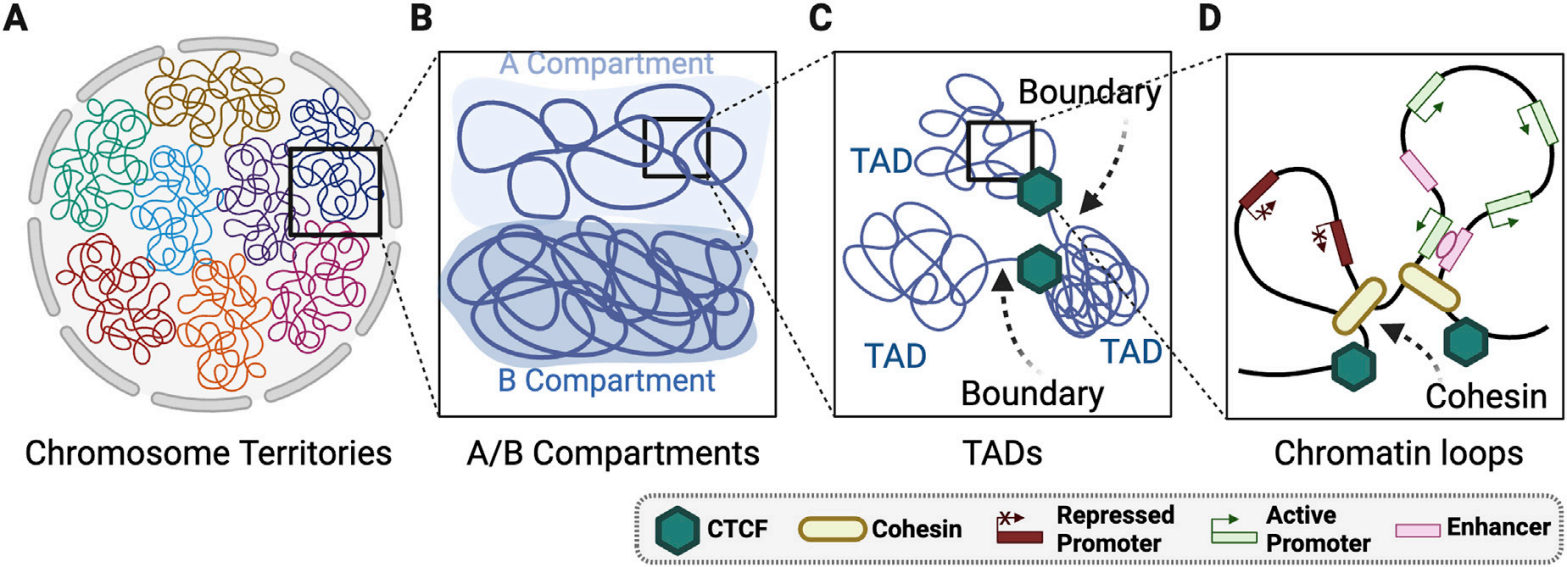
Layers of 3D genome organisation. **(A)** Whole chromosomes are spatially positioned into territories within the nucleus. **(B)** Chromosomes are organised globally into A and B mega base scale compartments within each chromosome territory. **(C)** Within A/B compartments there is regions organisation of highly self-interacting topologically associated domains (TADs) that are connected by TAD boundaries. **(D)** TADs are comprised of locally organised chromatin loops largely organised by cohesin and CTCF. Created with Biorender.com.

The fundamental functional and structural unit of chromatin are nucleosomes, which consist of a histone octamer wrapped twice with DNA (Giles and Taberlay, 2019; Luger et al., 1997). Arranging DNA within chromatin enables compaction of the genetic material within the nuclear volume and protects the DNA from the innate immune system and its cytoplasmic nucleases. The 3D structure of the genome is assembled through chromatin folding and the spatial organisation of large chromatin domains, creating distinct regulatory compartments for processes like transcription, DNA repair, and replication (Boettiger et al., 2016; Figures 1A–D). This physical compartmentalisation segregates transcriptionally active and inactive regions of the genome and is critical for establishing and maintaining replication timing (Bonev and Cavalli, 2016; Marchal et al., 2019). The 3D structure of the genome is assembled by architectural proteins that include CCCTC-binding factor (CTCF) and the structural maintenance of chromosomes (SMC) proteins within the cohesin and condensin complexes. These proteins promote the formation of chromatin loops and provide barrier elements that insulate genomic regions (Boettiger et al., 2016; Bonev and Cavalli, 2016; Phillips-Cremins et al., 2013; Pombo and Dillon, 2015; Rajderkar et al., 2023; Rao et al., 2014; Schalbetter et al., 2017; Sofueva et al., 2013). Despite our understanding of the molecular determinants of genome architecture there remains many unknowns regarding the biophysical principals of spatial genome compartmentalisation.

The hierarchical levels of 3D genome compartmentalisation are defined in interphase nuclei using chromosome conformation capture sequencing technologies such as Hi-C (Dekker et al., 2002; Lieberman-Aiden et al., 2009; Rao et al., 2014) (Figures 1A–D). Chromosomes occupy distinct non-overlapping nuclear territories (Figure 1A) (Hubner and Spector, 2010) classified into multi-megabase (Mb) scale A and B compartments (Figure 1B). The A compartments are transcriptionally active, gene-rich, preferentially located in the nuclear interior, and replicated in early S-phase (Lieberman-Aiden et al., 2009). Whereas B compartments are transcriptionally inactive, gene-poor, heterochromatic, and associated with late S-phase replication (Lieberman-Aiden et al., 2009). A/B compartments are further grouped into A1-A2, and B1-B4, which are linked to distinct genomic features (Lieberman-Aiden et al., 2009). For example, A1 compartments are associated with nuclear speckles (Chen et al., 2018), B1 compartments with polycomb bodies (Rao et al., 2014), and B2 and B3 compartments with lamin associated domains (LADs) (Rao et al., 2014; van Schaik et al., 2020).

Within the large-scale A/B compartments are smaller topologically associating domains (TADs) that consist of two main features: a highly self-interacting domain and boundary elements that restrict interactions between distinct TADs (Figure 1C; Dixon et al., 2012; Pombo and Dillon, 2015; Rao et al., 2014). TADs are largely, but not entirely, stable between cell types (Criscione et al., 2016). Hi-C methods now produce 3D genome maps of sufficient resolution to define nested subTADs within larger TADs. SubTADs are structurally akin to TADs but exhibit weaker boundaries and remain functionality undefined (Norton et al., 2018). Within TADs and subTADs are chromatin loops regulated by cohesin and CTCF (Figure 1D)(Szabo et al., 2020). Cohesin-regulated loop extrusion generates chromatin interactions, whereas CTCF insulates TADs by reducing inter-TAD contacts (Szabo et al., 2020). At the DNA-histone interface within the chromatin loops, the 3D genome is influenced through nucleosome phasing regulated by ATP-dependent chromatin remodelers (Barutcu et al., 2016; de Dieuleveult et al., 2016; Giles et al., 2019). Collectively, 3D genome topology is a multilayered structure regulated locally though nucleosome positioning, regionally via chromatin looping and TAD domains, and globally through large scale organisation of A/B compartments.

In addition to regulating transcription, TADs also delineate discrete replication domains and constrain the spreading of DNA damage markers around DNA double-stranded breaks (DSBs) (Arnould and Legube, 2020; Dixon et al., 2012; Pope et al., 2014). Notably, the boundaries between TADs are enriched for replication origins, active transcription, and become strengthened after DNA repair (Emerson et al., 2022; Sanders et al., 2020). Compared to cell-type specific TAD boundaries, evolutionally stable TAD boundaries have increased sequence conservation, higher enrichment of house-keeping genes, and greater CTCF binding (McArthur and Capra, 2021). This suggests important biological functions are regulated within these inter-TAD regions. However, many questions about TAD functions remain unanswered. For example, there is only a limited understanding of how endogenous genetic variation, exogenous stress, and skeletal forces (e.g., actin and microtubules) contribute to active TAD dynamics or plasticity. Further, we have a limited understanding of how disrupted TAD structures impact genome stability.

The connection between 3D genome structure and transcription is extensively reviewed (Bonev and Cavalli, 2016; Han et al., 2024; Krijger and de Laat, 2016; Kumar et al., 2021; Schoenfelder and Fraser, 2019; van Steensel and Furlong, 2019). Here we focus instead on the 3D genome during mammalian cell cycle progression and the role of the 3D genome in DNA replication and repair. Specifically, we discuss key steps in the transition between interphase and mitosis that preserve 3D genome memory; the order of DNA replication; and the role of the 3D chromatin architecture in maintaining genome integrity after DNA damage.

## 3D genome and the cell cycle

The cell cycle is an ordered, continuous series of events that controls genome replication and chromosome segregation. (Matthews et al., 2022). For over two decades, it has been evident that the cell cycle impacts the dynamic properties of the 3D genome (Dekker et al., 2002). More recently, single cell Hi-C experiments have demonstrated that cell cycle dependent changes are a major contributor to the dynamic organisation of the genome during interphase (Nagano et al., 2017; Naumova et al., 2013). During interphase the locations of the borders that define TADs were unchanged, but their intensity/insulation is dynamic. Insulation cannot be measured in mitotic cells but reaches a maximum as interphase nuclear architecture is reestablished during G1 phase. As cells enter S-phase, insulation (a measure of chromatin contacts across TAD boundaries) declines and plateaus at its lowest point in mid-S phase through to G2. The loss of insulation during S-phase coincides with the timing of replication. Early replicating TAD boundaries lose insulation in early S-phase and mid-late S-replicating boundaries lose insulation in mid-late S-phase. These findings suggest that replication across TAD borders or neighboring regions disrupts their chromatin contacts (Nagano et al., 2017; Naumova et al., 2013). Conversely, the kinetics of interphase A/B compartmentalisation differs from TADs. A/B compartmentalisation is weakest during G1 and increases across the cell cycle reaching a maximum in G2 before mitotic chromosome condensation dramatically restructures the genome during mitosis (Nagano et al., 2017). This implies that TADs and A/B compartments have distinct functions in interphase. Studying the restructuring of the genome during mitosis has provided many valuable insights into the principles governing genome organisation.

### Compacting the 3D genome for mitosis

**FIGURE 2:**
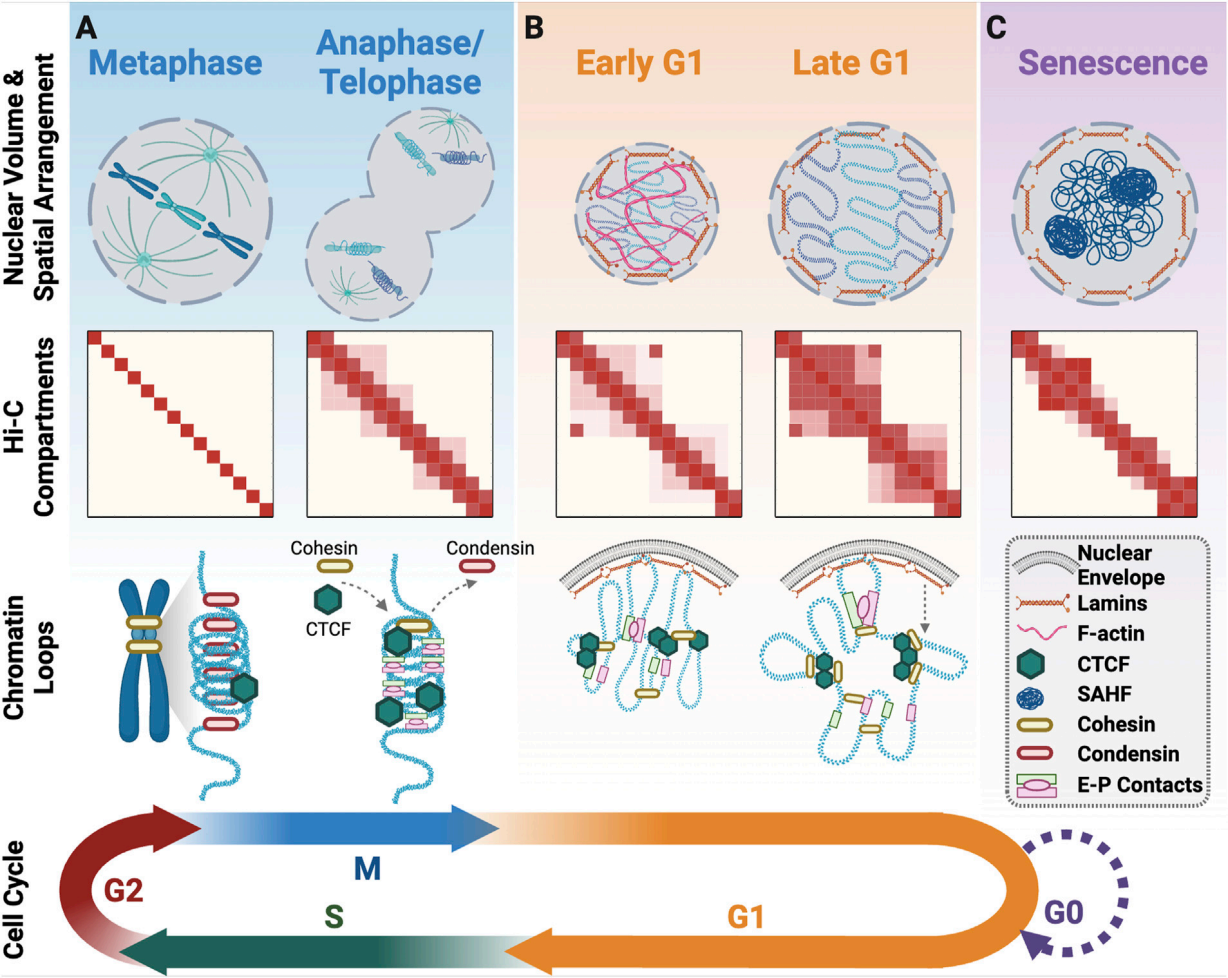
3D genome organisation upon cell cycle exit and re-entry. **(A)** Progression from metaphase to anaphase/telophase involves a change in the spatial positioning of DNA with the mitotic spindle pulling sister-chromatids in opposite directions from the metaphase plate. At this time, there is initial unwinding of chromatin and the beginning of interphase compartment formation. As cells exit mitosis, condensin is replaced with cohesin, and CTCF returns to all its binding sites for the formation of structural loops along with enhancer-promoter loops. **(B)** In Early G1 there is a burst of F-actin that facilitates nuclear volume expansion. A/B compartments and TADs become more defined across G1 and a subset of the mitotic enhancer-promoter loops are lost in favour of more cohesin/CTCF structural loops. **(C)** Senescent cells that have exited the cell cycle form senescence associated heterochromatin foci and have altered chromatin compartments. Created with Biorender.com.

The timely and ordered assembly of compact mitotic chromosomes during prophase is crucial for the faithful segregation in anaphase (Samejima et al., 2018). As cells enter mitosis the nuclear envelope breaks down and within the first minutes of prophase the A/B compartments and TADs are dismantled and little to no detectable signal remains in metaphase (Figure 2A; Gibcus et al., 2018; Nagano et al., 2017; Naumova et al., 2013; Zhang and Blobel, 2023). Loss of TADs and A/B compartments coincide with cohesin removal and condensin loading on the chromosome arms (Gibcus et al., 2018; Samejima et al., 2018; Waizenegger et al., 2000). Condensin I and II are the architectural scaffolds that control metaphase chromatin compaction and folding. During metaphase, when the chromosomes are highly condensed, chromatin contact frequency is high for regions within 10 Mb but rare for regions separated by more than 10 Mb (Naumova et al., 2013). Polymer modelling based on Hi-C observations suggests that mitotic chromosome condensation involves compaction of linear genome segments into 60 kilobase (kb) loops that are nested within larger 400 kb loops (Gibcus et al., 2018). Multiple condensin motors then create a helical “staircase” scaffold, which use loop extrusion to promote the axial compaction of the 400 kb loops into highly condensed mitotic chromatin (Figure 2A; Dey et al., 2023; Gibcus et al., 2018; Naumova et al., 2013).

Inactivating the condensin I and II complexes that coordinate the restructuring of chromatin during mitosis has provided valuable insights into their roles in this process (Gibcus et al., 2018). Depleting condensin complexes during interphase alters the assembly of mitotic chromatin, with specific mitotic A/B compartments partitioned, including the aggregation of facultative and constitutive heterochromatin (Zhao et al., 2024). While the TADs and local chromatin loops are still undetectable when the condensins are inactivated, cells are incapable of normal mitotic exit and they undergo mitotic slippage (Gibcus et al., 2018). This suggests that condensin complexes play key roles in both transitioning chromatin from interphase to mitotic structure and maintaining the mitotic chromatin structure until chromosome segregation is complete in telophase. Condensin is then replaced by cohesin during telophase when the chromatin structure begins to reform (Figure 2A) (Abramo et al., 2019; Zhang et al., 2019; Zhang H. et al., 2021). Temporal regulation of SMC complex switching from cohesin to condensin, and back, are thus key events in 3D genome transition at mitotic entry and exit. There is some evidence that other chromatin markers such as phosphorylation of histone 3 serine 10 (H3S10ph) and/or histone 3 lysine 9 di-methylation (H3K9me2) play a role in chromatin compaction (Castellano-Pozo et al., 2013; Lin et al., 2016; Wei et al., 1999). However, how these epigenetic modifications and their corresponding regulators alter the kinetics of TAD and A/B compartment dismantling for mitosis is yet to be determined.

### Organising the interphase 3D genome

Interphase chromosome positioning and spatial arrangement begins in telophase and continues into G1. In telophase, chromosomes rapidly decondense from rod-like mitotic structures into more spherical shapes, with chromatin destined for the A compartment expanding more rapidly than chromatin that will occupy the B compartment (Nagano et al., 2017). A/B compartment boundaries are established early, but long-range loop formation is a slow process that continues across the entirety of interphase (Abramo et al., 2019). In contrast, TAD structures form more rapidly beginning in telophase, with the establishment of TAD boundaries and their spatial positioning coinciding with the replication timing decision point (TDP), ∼3 h into G1 (Abramo et al., 2019; Dileep et al., 2015; Zhang et al., 2019). Architectural protein CTCF is dispensable for A/B compartment establishment after mitotic exit but is required for formation of short-range interphase chromatin loops (Zhang et al., 2019; Zhang H. et al., 2021). This is achieved by partial retention of CTCF on metaphase chromatin, coupled with rapid recovery of complete CTCF chromatin binding in anaphase/telophase (Figure 2A), to facilitate establishment of CTCF-cohesin dependent chromatin loops by early G1 (Zhang et al., 2019). Enhancer-promoter driven loops also form in anaphase and telophase (Zhang et al., 2019). These early enhancer-promoter loops are initially more prominent than CTCF-cohesin structural loops, indicative of their faster reconstruction upon mitotic exit (Figure 2A). As cells progress through G1, an increasing number of structural loops form and a subset of the early enhancer-promoter loops disappear (Figure 2B; Zhang et al., 2019). Acute CTCF depletion in mitosis restricts cohesin loop extrusion, resulting in persistence of the early enhancer-promoter loops into late G1 (Zhang H. et al., 2021). The function of early enhancer-promoter loops during late mitosis is unknown but their timing suggests a role in re-establishing genome architecture.

It is unclear how the genome architecture is preserved through cell division. Certain chromatin associated proteins and chromatin features have been proposed to act as a “mitotic bookmarks” to guide the assembly of genome organisation during interphase. The binding of RNA polymerase II (RNA pol II) to chromatin positions cohesin during the G1 transition (Zhang S. et al., 2021). Mitotic RNA pol II depletion can disrupt A/B compartments and the formation of TADs in G1, establishing a strong connection between transcription and 3D genome architecture (Zhang S. et al., 2021). Several transcription factors also possess mitotic bookmarking features that promote transcription restart upon mitotic exit (Blobel et al., 2009; Caravaca et al., 2013; Kadauke et al., 2012; Young et al., 2007; Zhao et al., 2011), how these transcription factors impact the 3D genome remains unclear.

Chromatin modifications could also have a role in establishing interphase genome organisation during mitotic exit. Bookmarking stem cell lineage-specific genes with histone 3 lysine 27 acetylation (H3K27ac) facilitates their rapid expression in early G1. This process is enhanced, but not strictly dependent on, the formation of interphase chromatin architecture (Pelham-Webb et al., 2021). H3K27ac also facilitates reformation of A compartments in condensin depleted mitotic chromatin (Zhao et al., 2024). The loss of mitotic H3K27ac, however, only impacts transcription, and not 3D genome organisation in G1-phase stem cells (Pelham-Webb et al., 2021). Therefore, H3K27ac contributes to redundant pathways that govern reestablishment of 3D genome architecture upon mitotic exit. H3K9me2 is an evolutionally conserved marker of heterochromatin that persists through mitosis and marks chromatin destined for the nuclear lamina (NL) (Poleshko et al., 2019). This helps rapidly establish genome organisation at the nuclear edge before cells exit mitosis (Poleshko et al., 2019), helping to reset peripheral chromatin structure for G1 re-entry.

### Nuclear lamina in 3D genome organisation

As established above, 3D genome organisation is established as the nuclear envelope reforms during mitotic exit. The nuclear lamina lines the inner surface of the nuclear envelope in eukaryotic cells and anchors heterochromatin domains to the nuclear envelope. The nuclear lamina (NL) is created through physical association of Lamin proteins (A-type and B-type) with the nuclear envelope and other factors. These interactions are essential for maintaining nuclear structure, regulating DNA replication, and controlling gene expression (Wong et al., 2022). The separation and organisation of heterochromatic Lamin associated domains (LADs) at the nuclear periphery is achieved by interactions between chromatin modifications, chromatin-binding proteins, and Lamins. Depletion of Lamin C (A-type) disrupts the association of heterochromatin LADs with the NL as cells enter interphase (Wong et al., 2021), and heterochromatin-associated histone modifications H3K9me2 and H3K9me3 mark spatially restricted LADs tethered to NL (Kind et al., 2013; Poleshko et al., 2019; van Schaik et al., 2020). Notably, a significant number of NL-LAD interactions are established within the first hour of nuclear envelope formation (Figure 2B). In terms of specific genomic regions, Telomere-proximal LADs attach to the NL rapidly, whereas centromere-proximal LADs accumulate more slowly (Crabbe et al., 2012; van Schaik et al., 2020). Contacts between NL-LADs shuffle stochastically with each cell cycle, but remain constrained, and stable throughout interphase (Jurisic et al., 2018; Kind et al., 2013; van Schaik et al., 2020). Why stochastic shuffling of NL-LADs occurs is unknown, but it indicates a degree of flexibility in genome organisation at this scale between cell cycles. The flexibility could be harnessed in response to environmental stresses where chromatin at the nuclear periphery is at higher risk to damage such as exposure to exogenous sources of DNA damage and nuclear envelope rupture.

### Nuclear filamentous actin in 3D genome organisation

As cells enter G1, filamentous actin (F-actin) supports nuclear structure by assisting with nuclear envelope reassembly, nuclear positioning, and chromatin organisation. F-actin within the nucleus mechanically supports daughter nuclei formation through nuclear protrusions and nuclear volume expansion (Figure 2B; Baarlink et al., 2017; Svitkina, 2018). F-actin driven nuclear expansion facilitates chromosome decondensation and diffusion within the first hour of G1, after which the nuclear F-actin is disassembled but its contribution to genome organisation persists into late G1 (Baarlink et al., 2017). Inhibiting F-actin polymerisation, but not branching, impairs post-mitotic nuclear expansion and chromatin organisation implicating F-actin generated intra-nuclear forces in the maintenance of genome architecture (Baarlink et al., 2017). It remains unclear, if nuclear F-actin directly interacts with chromatin to reshape the 3D genome, or if nuclear F-actin indirectly enables chromatin expansion by increasing the nuclear volume to establish the space required for interphase chromatin. F-actin’s role in genome organisation is also not strictly limited to nuclear reassembly in G1. F-actin can also reposition genomic regions for telomere maintenance, replication fork repair, and homologous recombination during S-phase and G2 (Harman et al., 2024; Lamm et al., 2020; Lamm et al., 2021; Schrank et al., 2018). Further investigation is needed to determine if F-actin has a similar role in nuclear organisation in non-cycling cells.

### Chromatin organisation during quiescence, senescence and differentiation

Quiescence is a reversible non-proliferating state caused by nutritional restriction and/or a reduction of growth factors. Transition between quiescence and active proliferation in adult hematopoietic stem cells is associated with 3D genome reorganisation (Takayama et al., 2021). Specifically, CTCF mediated 3D chromatin looping alters the transcriptional control of “stemness” genes and cell fate transitions (Takayama et al., 2021). Yeast also undergo 3D genome reorganisation in quiescence, during which condensin mediates chromatin condensation, and there are respective decreases and increases in centromere and telomere interactions (Rutledge et al., 2015). Embryonic stem cells (ESCs) use quiescence to retain plasticity to generate both embryonal and extra-embryonal cell types (Khoa et al., 2024). The extent, if any, of 3D genome reorganisation for this transition is unknown.

Senescence is a permanent halt to cell proliferation induced by cellular stress, including DNA damage and/or replicative ageing, or physiological differentiation into post-mitotic tissues including neurons, end stage B cells, or cardiomyocytes (Terzi et al., 2016). Both stress-induced and physiological senescence are associated with partial 3D genome reorganisation into senescence-associated heterochromatin foci (SAHF) (Pombo and Dillon, 2015; Shaban and Gasser, 2023; Terzi et al., 2016). Each SAHF is formed from a single chromosome and corresponds with reduced transcription (Figure 2C; Swanson et al., 2015; Terzi et al., 2016). Within the 3D genome of cells experiencing early replicative senescence, there are increases in long range chromatin interactions, CTCF clustering, and partial compartment switching (Zirkel et al., 2018). During late replicative senescence, short range chromatin interactions increase, as does TAD compartmental switching. TAD boundaries, however, remain conserved (Criscione et al., 2016).

Differentiation of neuronal stem cells into astrocytes, which are largely considered post-mitotic, also corresponds with changes in genome organisation, but TAD boundaries remain consistent (Sofueva et al., 2013). Depleting cohesin in astrocytes confers a global relaxation, but not an abolishment of TADs, suggesting other features maintain genome structure in differentiated post-mitotic cells (Sofueva et al., 2013). It is possible that 3D genome alteration in these cells results from LAD disruption, which is known to occur in senescent cells. In agreement, depleting Lamin B1 artificially alters the NL, resulting in heterochromatin reorganisation and SAHF formation (Sadaie et al., 2013; Shaban and Gasser, 2023). Additionally, neural progenitor cells in Down’s syndrome patients display 3D genome reorganisation coincident with LAD disturbance and the appearance of senescence hallmarks (Meharena et al., 2022). If 3D genome alteration is a cause or consequence of senescence, however, remains to be determined.

## DNA replication

DNA replication is the duplication of DNA for transmission of genetic information into daughter cells upon cell division. During DNA replication, genetic and epigenetic information stored in the DNA, histones and 3D structure of the genome needs to be preserved to maintain genome stability and cell identity. DNA replication begins from replication origins, then proceeds bidirectionally through the DNA in a semiconservative manner. Each replication fork synthesises a leading strand in the same direction as the fork progression and a lagging strand synthesised as 100-200bp discontinuous Okazaki fragments in the opposite direction that are joined by DNA ligase (Snedeker et al., 2017).

In eukaryotes, DNA replication is performed according to a strict replication timing (RT) programme that coordinates the deployment of the replication machinery across the genome through the coordinated activation of selected replication origins. RT is observed at the megabase scale (Mb) with “timing domains” replicating via clusters of replication origins, that fire at similar times during S-phase. The regulation of origin firing by the RT programme ensures the genome is copied in its entirety, once and only once, per cell cycle in a timely manner preventing genetic mutations and larger structural chromosome rearrangements that have the potential to drive tumorigenesis or trigger cell death (Hu and Stillman, 2023; O’donnell et al., 2013).

### Replication timing and the 3D genome

**FIGURE 3:**
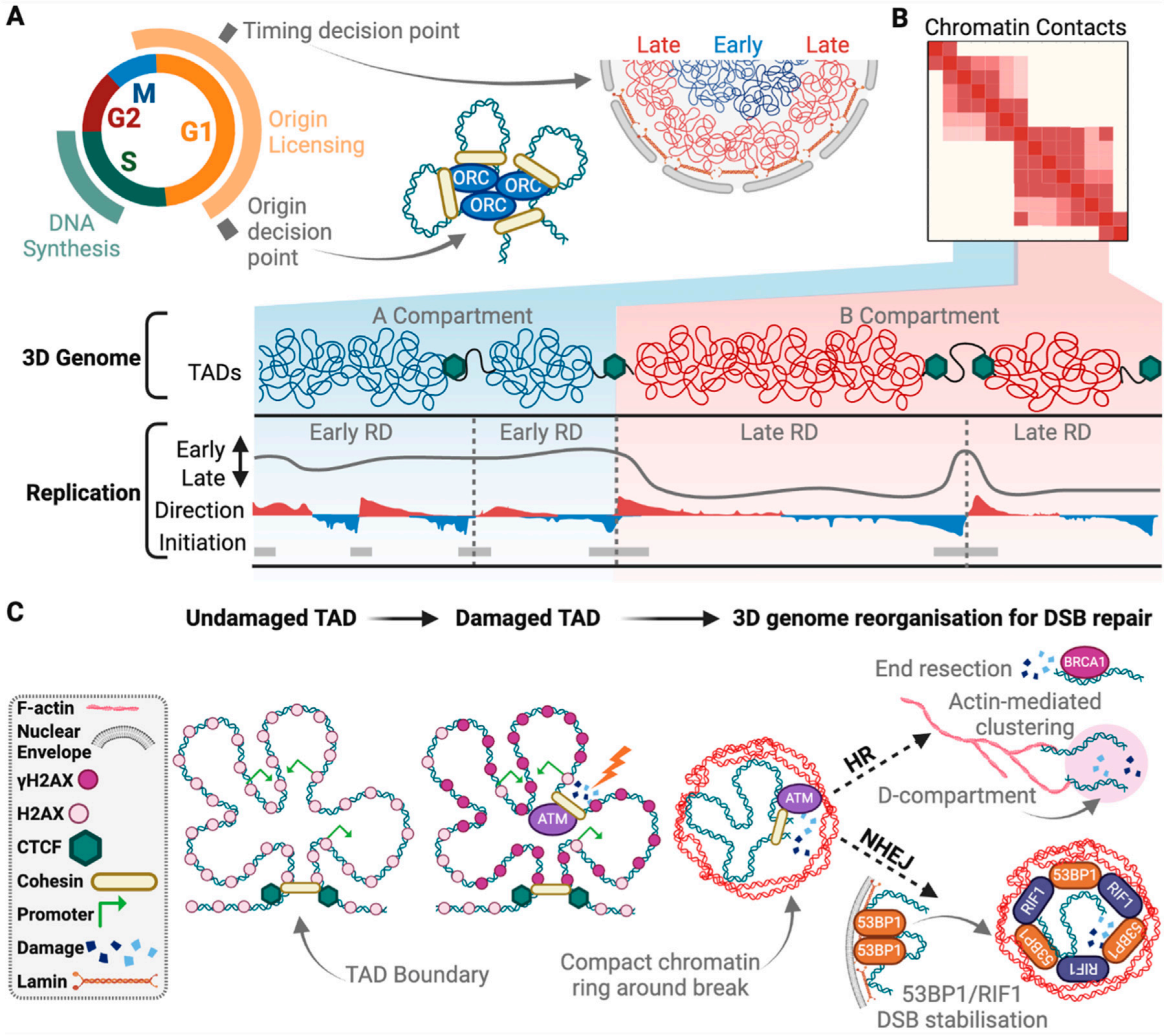
The 3D genome in DNA replication and repair. **(A)** The timing decision point occurs early in G1, during which, early replicating chromatin is positioned near the centre of the nucleus, while the late replicating DNA is nearer to the nuclear boundary. Late G1 is the origin decision point. Here chromatin structural loops bring together licenced origins to be activated in S-phase and initiate DNA synthesis occurs. **(B)** Within “A” compartments are early replicating domains (RD) with higher density of replication initiation sites compared to late RDs in “B” compartments. Direction of replication measured by OK-seq and indicates forks moving to the right (red) or left (blue). **(C)** Upon DSB induction γH2AX signalling is spread within the damaged TAD via chromatin contacts, except for at active gene promoters, and bordered by CTCF binding. Local 3D genome structure is rearranged to form open chromatin around the break site (blue), surrounded by a compact chromatin boarder (red). For NHEJ, 53BP1 is released from LADs and stabilises the break site for repair with RIF1, while for HR, BRCA1 promotes end resection that signals to actin for break clustering into a D-compartment for repair. Created with Biorender.com.

The regulatory mechanisms that control replication timing are not well understood, but significant progress has been made by studying the 3D organisation of timing domains. The pattern of RT domains across the genome closely resembles the long-range genome interaction maps, providing strong evidence that the 3D organisation of the genome is important for determining replication timing (Figures 3A, B; Ryba et al., 2010). RT is also correlated with nuclear positioning, chromatin accessibility and transcription (Dileep et al., 2015; Pope et al., 2014). Regions that replicate early during S-phase are positioned within transcriptionally active TADs, in the A compartment. In contrast, late-replicating regions are located within transcriptionally silent TADs and LADs, enriched with heterochromatin histone modifications, and in the B compartment located at the nuclear and nucleolar periphery (Jackson and Pombo, 1998; Marchal et al., 2019). The replication timing programme is evolutionarily conserved and changes during cell differentiation, coinciding with the remodeling of the 3D genome (Marchal et al., 2019). During differentiation, loci that shift from early to later replication, frequently re-localise to the nuclear periphery (Hiratani et al., 2008). Despite these observations, the precise biological relevance of RT has been challenging to address. It has been difficult to determine whether RT is a consequence of genome organisation, chromatin structure, or gene expression, and whether it plays a direct role in these processes.

Recent research into the molecular determinants of RT and its regulation during development has provided strong evidence that the spatiotemporal coordination of replication is essential for preserving the epigenome and shaping the 3D structure of the genome (Vouzas and Gilbert, 2023). Genetic dissection of ‘Rap1 interacting factor 1’ (RIF1), a key regulator of RT, has provided much-needed insights into the links between RT and genome organisation. RIF1 was first identified in yeast as a regulator of telomere length and transcriptional silencing (Hardy et al., 1992). Subsequent studies revealed its critical roles in replication timing, genome organisation, epigenome preservation, and DNA repair (Alavi et al., 2021; Chen and Buonomo, 2023). RIF1’s interaction with Protein Phosphatase 1 (PP1) is critical for its roles in RT and genome organisation. However, expression of RIF1 mutants that cannot bind PP1 can partially restore RT, suggesting that RIF1 may have PP1-dependent and independent roles in RT regulation.

The recruitment of RIF1 to late replicating chromatin provides the most probable locations of its critical PP1 targets (Gnan et al., 2021; Hiraga et al., 2017; Sukackaite et al., 2017). RIF1’s ability to bind to PP1 may partially contribute to replication timing by counteracting origin firing at late replicating regions by opposing the ‘DBF4-dependent kinase’ (DDK)-mediated phosphorylation of the minichromosome maintenance (MCM) complex (Alver et al., 2017; Hiraga et al., 2014; Mattarocci et al., 2014). In humans, DDK inhibition suppresses DNA synthesis within late replicating genomic regions via a process that requires RIF1 and ‘Ataxia telangiectasia and Rad3 related’ (ATR) (Jones et al., 2021; Rainey et al., 2020). The timing-specific differences observed in these studies suggest that the order of replication may in part be achieved through varying the requirements for DDK activity. Late replicating regions rely more heavily on DDK activity to overcome RIF1 and ATR-dependent suppression of origin firing. In contrast, early replicating domains, where RIF1 is absent, require minimal DDK activity for origin firing. In fact, cyclin-dependent kinase (CDK) activity has been shown to compensate for the inhibition of DDK during early S-phase (Suski et al., 2022).

Investigating RIF1’s role in genome organisation is also providing insights into the broader requirements for RT. During G1 phase, RIF1 participates in the re-establishment of the 3D nuclear architecture by restricting interactions between replication domains with similar replication timing (Foti et al., 2016). Loss of RIF1 leads to widespread changes in replication timing and the distribution of histone modifications, followed by alterations in genome organisation and limited alterations in gene expression (Klein et al., 2021). RIF1’s role in genome organisation is highly sensitive to RIF1 dosage, whereas its role in RT requires complete loss of function. Partial loss of RIF1 (haploinsufficiency) disrupts chromatin organisation without affecting RT, demonstrating that its genome organisation roles can be uncoupled from RT phenotypes. The different phenotypic outcomes between partial and complete loss of RIF1 suggests that genome organisation does not strictly define RT (Chen and Buonomo, 2023; Gnan et al., 2021). For example, MCM6 depletion causes widespread changes in RT without impacting genome organisation (Peycheva et al., 2022). Moreover, directly disrupting genome organisation via cohesin or CTCF depletion does not impact RT (Oldach and Nieduszynski, 2019; Sima et al., 2019).

### Replication machinery and the 3D genome

While not influencing RT, cohesin instead impacts DNA replication by influencing the processivity of replication machinery and the positioning and selection of replications origins (Figure 3A). Cohesin can act as a physical obstacle to the replisome, and conditions that stabilise chromatin-bound cohesin or disrupt sister chromatid cohesin lead to reduced replication fork speed (Carvajal-Maldonado et al., 2019; Morales et al., 2020; Terret et al., 2009). The cohesin complex is enriched at a highly efficient subset of replication origins that are positioned at TAD boundaries (Figures 3A, B) (Courbet et al., 2008; Emerson et al., 2022; Giles et al., 2022; Guillou et al., 2010; MacAlpine et al., 2010). However, the mechanisms underlying how replication origins are positioned at TAD boundaries is a matter of debate, with multiple models proposed to explain this phenomenon. One possibility is that cohesin-mediated loop extrusion could slide the pre-replicative MCM complexes along chromatin until they reach an appropriate CTCF boundary element (Emerson et al., 2022). An alternative model is that MCM complexes may act as physical barriers themselves that restrict cohesin translocation, halting or pausing loop extrusion at replication origins (Dequeker et al., 2022). This model suggests that inactive MCM complexes are loaded onto chromatin in G1 to establish the 3D structure of the genome. Finally, loading of cohesin by the MCM complex and DDK during sister chromatid cohesin could also explain the enrichment of cohesin at origins but these sites would not necessarily be positioned at TAD boundaries (Zheng et al., 2018). Resolving how replication origins are assembled at TAD boundaries will address fundamental questions about the mechanisms required for origin positioning, selection, and the role of the 3D genome structure in this process.

### DNA replication and epigenome maintenance

Disrupting RT interferes with the maintenance of the epigenome (Klein et al., 2021). Precisely how RT contributes to the accurate distribution of histone modifications is unclear, but an exciting possibility is via the recycling of parental histone. During replication, histone chaperones are recruited to the replisome to couple DNA unwinding with the disassembly of parental nucleosomes (Groth et al., 2007; Yang et al., 2016). Modified parental histones are recycled and combined with newly synthesised naive histones during chromatin assembly behind the replication fork. The recycling of parental histones provides positional information for chromatin readers and writers that ensure the histone modifications are not diluted during replication (Flury and Groth, 2024). Recent methods for analysing the distribution of parental histones on newly replicated DNA are providing valuable mechanistic insights into how the replisome directs epigenetic inheritance (Escobar et al., 2021; Petryk et al., 2021). These techniques include a CRISPR-biotinylation system to track parental nucleosome segregation at single loci and chromatin occupancy after replication (ChOR)-seq (Escobar et al., 2019; Petryk et al., 2021). The symmetrical segregation of parental histones onto the leading and lagging strands during DNA replication is crucial for maintaining the epigenome in daughter cells. Asymmetric segregation of parental histones can suppress differentiation in mouse embryonic stem cells (ESCs) (Wenger et al., 2023) and promote tumour growth and invasion (Tian et al., 2023). The asymmetric segregation of parental histones into daughter cells may also contribute to establishing different cell fates during development through asymmetric cell divisions (Wooten et al., 2020).

Molecular mechanisms that coordinate the recycling of parental nucleosomes have been identified with MCM2 and the fork protection complex protein Mrc1/Claspin recycling parental H3-H4 tetramers. Mrc1 is capable of shuttling parental H3-H4 tetramers to leading or lagging strands (Charlton et al., 2024). The histone chaperone, nucleophosmin (NPM1) is also critical for nucleosome segregation within late replicating facultative heterochromatin. NPM1 binds polycomb repressive complex 2 (PRC2) and MCM2 to ensure H3K27me3 is maintained within the repressed chromatin domains (Escobar et al., 2022). It is unclear how the 3D organisation of the genome and the timing of replication ensure the accurate recycling of parental nucleosomes. Histone chaperones may be tightly regulated or recruited to replication forks within particular TADs at specific times in S-phase (Escobar et al., 2021; Escobar et al., 2019), which raises the question of how temporal recruitment could be achieved. Future studies focusing on the regulation and recruitment of histone chaperones in combination with Hi-C, ChOR-seq and Repli-seq, will provide further insights into how replication ensure accurate inheritance of the epigenome.

### Genome organisation and replication during development

Early-stage embryos provide a unique setting to study the establishment of genome architecture and RT. Technical advances in single-cell Repli-Seq (scRepli-Seq) have enabled high-resolution profiling of replication states within individual S-phase cells (Dileep and Gilbert, 2018; Takahashi et al., 2019). scRepli-Seq has been used to examine the earliest embryonic divisions revealing that nuclear organisation is established in the zygote before RT, which appears at the 4-cell stage (Nakatani et al., 2024; Takahashi et al., 2024; Xu et al., 2024). These findings demonstrate that RT is established prior to embryonic genome activation and operates independently of transcription. Prior to the establishment of RT, cells display signs of replication stress with slow fork speed, fork stalling and fork collapse during the first S-phase in 1-cells embryos (Palmerola et al., 2022). Chromosome breaks can be observed at the 4- to 8-cell divisions with breakpoints typically localised to late-replicating gene-poor regions (Xu et al., 2024). By the 8-cell stage, RT is established and fork speed increases and chromosome aberrations are reduced. These findings suggest replication stress during the early embryonic divisions when the 3D genome, RT, and epigenetic regulation are being established could be a source of genomic instability that contributes to germline mutations (Takahashi et al., 2024). Investigating the cause of replication stress and the tolerance pathways that suppress and repair DNA damage in these early embryonic divisions will be critical for understanding its contribution to *de novo* mutations and improving *in vitro* fertilisation techniques.

## DNA repair

Efficient, high-fidelity DNA repair is critical to maintain genome integrity. Genomes endure thousands of lesions per day, from endogenous sources such as replication errors, and exogenous threats such as toxins or radiation. DSBs are the most dangerous form of DNA damage and can arise from ionizing radiation (IR), X-rays, ultraviolet (UV) light, reactive oxygen species, replication stress, toxic chemicals, and/or aberrant enzyme activity (Chang et al., 2017; Jackson and Bartek, 2009; Tubbs and Nussenzweig, 2017). DSBs also occur during specific physiological processes including V[D]J recombination, immunoglobulin class-switching, and meiotic sister chromatid exchange (Jackson and Bartek, 2009). Unrepaired or mis-repaired DSBs are a major source of genome instability with potential catastrophic consequences, including cell death and oncogenesis (Jackson and Bartek, 2009; Szmyd et al., 2025; Tubbs and Nussenzweig, 2017).

The cellular response to DSBs is initiated through the DNA damage response (DDR). Following genome damage, the master kinases ‘ataxia-telangiectasia mutated’ (ATM), ATR, and/or ‘DNA-dependent protein kinase ‘(DNA-PK) are activated to coordinate cell cycle arrest, nuclear cytoskeleton function, modulation of the 3D genome, and DNA repair (Arnould et al., 2023; Blackford and Jackson, 2017; Caron et al., 2015; Jackson and Bartek, 2009; Menolfi and Zha, 2022). At the chromatin level, ATM, ATR, and DNA-PK rapidly phosphorylate the modified histone variant H2AX on ser139, termed γ-H2AX when phosphorylated, in the break adjacent chromatin. γ-H2AX modification is an early step in the DDR and establishes the early alterations to damaged chromatin that potentiate eventual DNA restoration.

After initial DDR activation, DSBs are repaired through four cell-cycle dependent repair pathways. Non-homologous end joining (NHEJ) functions through the cell cycle and is the primary, and potentially only, DSB repair pathway available in G1 phase (Branzei and Foiani, 2008; Guirouilh-Barbat et al., 2008; Hustedt and Durocher, 2016; Rothkamm et al., 2003). Microhomology mediated end joining (MMEJ), single strand annealing (SSA), and homologous recombination (HR) function in S and G2 (Branzei and Foiani, 2008; Chang et al., 2017; Hustedt and Durocher, 2016; Rothkamm et al., 2003). MMEJ also has mitotic repair activity (Brambati et al., 2023; Wang et al., 2018) and may function at low levels in G1 (Truong et al., 2013; Xiong et al., 2015). In addition to cell cycle stage, several other factors influence repair pathway choice; including DNA end resection at the break site, the local chromatin state, transcriptional activity in the effected genomic region, and DNA mobility (Aymard et al., 2014; Carvalho et al., 2014; Pfister et al., 2014; Symington and Gautier, 2011).

### The 3D genome creates functional regional compartments for DNA repair

Chromatin A/B compartments are respectively linked to euchromatin and heterochromatin. Euchromatin A compartments have a higher incident of endogenous breaks from enzyme induced damage (Canela et al., 2016; Lensing et al., 2016), whereas heterochromatin B compartments are more likely to obtain breaks from UV irradiation, but confer protection from ionising radiation (Fortuny and Polo, 2018; Takata et al., 2013). Chromatin in both A and B compartments has been reported as susceptible to replication stress induced damage (Crosetto et al., 2013; Fortuny and Polo, 2018). The kinetics of γH2AX foci formation after DNA damage differs between euchromatin and heterochromatin. γH2AX foci form immediately after damage in euchromatin but have a delayed response in heterochromatin (Natale et al., 2017). When multiple DSBs are induced by expression of the AsiSI restriction endonuclease, there is an approximate 15% switch of B to A compartments (Zagelbaum et al., 2023). Therefore, while genome breaks confer some large-scale changes to 3D genome architecture, most A/B compartments remain unaffected.

At a regional level, TAD structure has a strong connection to γH2AX spreading around DSBs. Following break induction, γH2AX generally decorates 1–2 Mb of chromatin in a bidirectional but asymmetric fashion around the DNA lesion. γH2AX spreading is largely, but not exclusively, constrained to the affected TAD (Figure 3C)(Arnould and Legube, 2020; Collins et al., 2020; Iacovoni et al., 2010). Re-creating a break in the identical location results in similar TAD-constrained γH2AX spreading (Arnould and Legube, 2020; Arnould et al., 2021). Shifting break location to a different region of the same TAD redistributes γH2AX around the break but retains γH2AX within the affected TAD (Arnould et al., 2021). Interestingly, the spatial spreading of γH2AX occurs via 3D chromatin interactions from the DSB site, not linearly through the TAD (Figure 3C)(Arnould and Legube, 2020; Caron et al., 2012; Collins et al., 2020). Additionally, the data suggest that TAD boundaries strengthen when a nuclease-induced DSB occurs, which is dependent on ATM (Arnould et al., 2023). This likely insulates the TAD and constrains γH2AX from spreading to adjacent TAD structures. In agreement, disrupting TAD boundaries increases γH2AX spreading into neighbouring TADs (Collins et al., 2020). TADs thus compartmentalise DDR activity within the regional 3D genome structure.

53BP1 is a DDR marker that also localizes within TAD structure following DSB induction (Ochs et al., 2019). This is consistent with the strong overlap between 53BP1 and γH2AX immunofluorescence foci following damage induction. Super resolution imaging demonstrated that 53BP1 forms nanodomains that alternate with RIF1 in a ring structure at DSBs (Figure 3C; Ochs et al., 2019). Each nanodomain is occupied by an individual TAD, and this structure is proposed to safeguard genome integrity (Ochs et al., 2019). Depleting RIF1, or expressing a mutant 53BP1 that prevents RIF1 recruitment, disrupts the cytological ring structure, confers aberrant spreading of repair proteins, and promotes hyper-resection of DNA ends (Ochs et al., 2019). This is phenocopied by cohesin depletion, suggesting cohesin and RIF1 cooperate to preserve the 3D genome structure and promote NHEJ (Ochs et al., 2019).

The tight connection between TADs, γH2AX, and 53BP1 foci raises the question of how DSBs in different elements of 3D genome architecture, such as TAD boundaries vs. domains, influence DNA repair pathway choice and genome stability. For instance, DSBs buried within TADs may impair repair factor availability for HR. There is no concordance between TADs and linkage disequilibrium (Whalen and Pollard, 2019), implying that physiologically induced breaks for non-sister chromatin recombination in meiosis do not occur at TAD boundaries. In contrast, DSBs are enriched at TAD boundaries during neuronal degeneration, leading to reduced TAD definition and fewer chromatin loops (Dileep et al., 2023). This suggests that DSBs in TAD boundaries are disruptive to 3D genome structure and are likely more detrimental to genome function.

### DSB spatially reorganise local 3D genome structure

The local chromatin structure surrounding DSBs is spatially modified after break induction. Laser mediated DNA damage triggers immediate compaction around the DSB (Lou et al., 2019). This is followed shortly thereafter by chromatin decompaction at the lesion and establishment of a ring of compact chromatin surrounding the break (Figure 3C; Burgess et al., 2014; Lou et al., 2019; Luijsterburg et al., 2016). Following break induction, PARP1 recruits the ATP-dependent chromatin remodeller CHD2 to the lesion, triggering chromatin expansion and deposition of the histone variant H3.3 (Luijsterburg et al., 2016). Chromatin compaction status at DSBs is further governed by ATM and the downstream ubiquitinase RNF8. RNF8 and its target RNF168 regulate large scale ubiquitinylation of chromatin at DSB sites to promote repair (Doil et al., 2009; Feng et al., 2024; Krais et al., 2021; Mattiroli et al., 2012; Stewart et al., 2009). Inhibiting ATM or RNF8 disrupts the compact chromatin border established around DSBs (Lou et al., 2019). Creating a compacted border surrounding an open lesion likely constricts 53BP1 and γH2AX to the surrounding TAD, and prevents transcriptional interference at the break, whilst leaving the lesion accessible to repair factors (Lou et al., 2019; Natale et al., 2017).

In yeast, DSBs commonly move to the periphery for repair (Nagai et al., 2008; Oza et al., 2009). This, however, is not readily observed in mammalian cells except for rDNA and alpha satellites (Harding et al., 2015; Tsouroula et al., 2016; van Sluis and McStay, 2015). Chromatin does, however, mobilise in mammalian cells to cluster DSBs and compartmentalise the genome for repair. DSB clustering is more prominent in A compartments and enriched during G1 repair of breaks in active genes (Arnould et al., 2023; Aten et al., 2004; Aymard et al., 2017). Clustering has been hypothesised to delay NHEJ, which may be detrimental to active genes, and promote HR later in the cell cycle (Aymard et al., 2017). 3D genome architecture is altered as DSB cluster to form “D compartments” (Figure 3C; Arnould et al., 2023). D-compartments generally have a strong correlation with γH2AX, contain active histone marks and upregulated genes containing R-loops (Arnould et al., 2023). R-loops are three-stranded RNA-DNA hybrids formed during transcription, with active roles in gene regulation, class-switching recombination and DNA replication in physiological conditions, but can also cause DNA damage and genome instability (Petermann et al., 2022; Sollier and Cimprich, 2015). R-loops can be structural barriers for cohesin (Wulfridge et al., 2023; Zhang et al., 2023) and may play a role in limiting cohesin-mediated loop extrusion in DSB clustering.

The rearrangement of 3D genome structure for DSB clustering appears to be potentiated through mechanical forces (Lamm et al., 2021). A series of papers identified that nuclear-based actin polymerisation, mediated by the F-actin nucleator Arp2/3, its activator WASP, and actin regulators Formin-2 and Spire-1/2, function in DSB and replication stress repair (Figure 3C; Belin et al., 2015; Schrank et al., 2018; Zagelbaum et al., 2023). WASP co-localises with γH2AX in both G1 and G2, whereas Arp2/3 is only found at DSBs in G2 (Schrank et al., 2018). Restriction of Arp2/3 binding at breaks to G2, may be a feature that delays active gene repair until a homologous chromosome is available. Nucleus-specific actin also functions in replication stress repair, which is mediated by HR factors, consistent with actin related forces functioning in recombinational DNA restoration (Lamm et al., 2020).

Microtubule forces are also implicated in some specific DNA repair outcomes (Kim, 2022). Partially depleting the telomere protective factor TRF2 promotes DDR-positive telomeres that remain resistant to NHEJ-dependent covalent ligations (Cesare et al., 2013; Van Ly et al., 2018). These DDR-positive telomeres cluster within nuclear regions co-stained for 53BP1 and γH2AX immunofluorescence (Cesare et al., 2013; Timashev et al., 2017), and both ATM and 53BP1 are required for telomere mobility in the absence of repair (Dimitrova et al., 2008). Clustering is therefore a function of the upstream DDR, and not necessarily dependent upon repair. NHEJ-dependent ligation of telomeres completely lacking TRF2 is mediated by microtubule forces, potentiated to the nucleus through the transnuclear membrane LINC complex (Lottersberger et al., 2015). This suggests that cytoskeletal forces originating from the cell body can potentiate chromatin mobility in the nucleus. There is an emerging understanding of potential roles for microtubules in DNA repair (Gerlitz et al., 2013; Lerit and Poulton, 2016; Ma et al., 2021; Oshidari et al., 2018; Shokrollahi et al., 2024). At present, it is unclear if microtuble forces alter the 3D genome compartments or TAD structures.

DSB clustering may compartmentalise the genome for efficient repair at the potential cost of chromosome aberrations (Arnould et al., 2023; Aten et al., 2004; Aymard et al., 2017). Translocations in cancer are enriched in D-compartments (Arnould et al., 2023) and inhibiting the F-actin nucleator Arp2/3 both prevents B to A compartment switching after DSB and reduces translocation risk (Zagelbaum et al., 2023). Translocations, however, still occur in Arp2/3 inhibited cells from mis-repaired NHEJ, indicating that translocations can occur independent of DSB clustering (Zagelbaum et al., 2023). It is possible this stems from microtubule forces, consistent with the NHEJ-telomere telomere-telomere fusions observed in cells completely devoid of TRF2 (Lottersberger et al., 2015).

### Chromatin architectural proteins facilitate DSB repair

Chromatin architectural proteins play important roles in both TAD structure and DSB clustering. CTCF is recruited to DSBs within seconds of lesion creation. This is mediated through PARylation and the CTCF zinc finger 4–6 (ZNF4-6) domain (Han et al., 2017; Hilmi et al., 2017). Following recruitment, CTCF flanks the DSB and creates a boundary to confine γH2AX foci. This both preserves 3D genome organisation and promotes efficient HR repair (Han et al., 2017; Hilmi et al., 2017; Lang et al., 2017; Natale et al., 2017). Deleting ZNF4-6, or chemically inhibiting PARylation, prevents CTCF recruitment and increases IR susceptibility (Han et al., 2017; Lang et al., 2017). CTCF depletion also increases chromosomal instability, end-to-end fusions, DNA breaks, and apoptosis (Hilmi et al., 2017; Lang et al., 2017).

CTCF directly interacts with DDR and HR factors, including MDC1, Rad51 and Ago2, to promote repair (Lang et al., 2017). Roles for CTCF in HR include BRCA1-independent BRCA2 recruitment and Rad51 foci formation (Hilmi et al., 2017; Lang et al., 2017). Notably, while CTCF colocalises with 53BP1, it does not contribute to NHEJ, nor effect 53BP1 recruitment or clearance (Hilmi et al., 2017; Lang et al., 2017). Consistent with CTCF promoting HR, depleting CTCF increases NHEJ incidence 34% while reducing HR efficiency by 47% (Lang et al., 2017). CTCF, and presumably chromatin looping, therefore, helps maintain genome stability by promoting effective HR.

Cohesin also functions in DSB repair by promoting loop extrusion around break sites but does so independent of CTCF (Arnould et al., 2021). In yeast, cohesin spreads extensively around DSBs (Oum et al., 2011; Strom et al., 2004; Unal et al., 2004), whereas cohesin spreading in mammals is restricted to the 2–5 kb adjacent to the break (3C)(Arnould et al., 2021; Caron et al., 2012). Loading cohesin at mammalian DSBs requires NIPBL, ATM kinase, and the repair factor MRE11, all of which have a similar binding distribution around break sites (Arnould et al., 2021; Caron et al., 2015; Dileep et al., 2023). Cohesin binds either side of the DSB and promotes one-sided loop extrusion to increase chromatin contacts surrounding the break site. This continues until arrested by a chromatin boundary (Arnould et al., 2021; Arnould et al., 2023). ATM inhibition abolishes cohesin-mediated loop extrusion at breaks, while DNA-PKcs inhibition increases it, potentially skewing repair pathway choice (Arnould et al., 2023; Caron et al., 2015). As nucleosomes that contain H2AX pass cohesin during this loop extrusion, H2AX is rapidly phosphorylated to become γH2AX (Arnould et al., 2021). The exception is H2AX in active gene promoters which are not phosphorylated to maintain transcription (Figure 3C; Caron et al., 2012). Cohesin depletion increases the intensity of γH2AX signal in ChIP-chip experiments, corresponding with a transcription downregulation within the γH2AX demarcated domain (Caron et al., 2012).

Cohesin functions vary between break location. In some genomic locations, cohesin confines γH2AX spreading, while in others γH2AX domains spread independent of cohesin (Caron et al., 2012). It remains unclear if cohesin is a driver or passenger to this process, and the potential impact of chromatin context, nucleosome positioning, and/or the cell cycle on cohesin-mediated repair functions. Cohesin also limits the mobility of DSBs by tethering broken ends to protect them from spontaneous end joining. Following DNA replication in S and carrying into G2, cohesin links the two sister chromatids to repress distant end joining and promote repair through the adjacent chromatid (Gelot et al., 2016). However, during replication stress, cohesin increases end joining through NHEJ and MMEJ, which can result in chromosome fusions (Gelot et al., 2016). Cohesin therefore mediates loop-extrusion to promote repair activation and protects transcriptional output through γH2AX exclusion at promoters. Cohesin also supresses DSB mobility by tethering broken ends to protect against deletions, inversions, translocations and chromosome fusions mediated by unscheduled end joining.

Lamins impact DNA repair, though little mechanistic detail is known. Lamin B1 controls the release of 53BP1 after DSB induction (Figure 3C; Etourneaud et al., 2021). Moreover, Lamin B1 overexpression also impedes 53BP1 recruitment to DSBs, impairs NHEJ, and results in persistent breaks and increased damage sensitivity (Dileep et al., 2023; Etourneaud et al., 2021). The related factor Lamin A also impacts 53BP1 and NHEJ. Lamin A is required for the processing of dysfunctional telomere heterochromatin by NHEJ through stabilisation of 53BP1 (Gonzalez-Suarez et al., 2009), and depleting of Lamin A results in genome instability through telomere shortening (Gonzalez-Suarez et al., 2009). Further work is required to better understand the role of Lamins and LADs in chromatin organisation for DNA repair. A deeper exploration of these dynamics will shed light on the broader relationship between nuclear architecture and DNA repair.

### Radiation-induced DNA damage

Most studies investigating the 3D genome in DNA repair utilise defined DNA breaks induced by CRISPR or other nucleases. Little is known about how simultaneous induction of multiple stochastically positioned different types of breaks, as occurs following irradiation, effects 3D genome architecture. One study did investigate 3D genome structure in G1/G0 arrested BJ-5ta foreskin fibroblasts and cycling GM12878 lymphoblastoid cells following ionising radiation (IR) (Sanders et al., 2020). At 24 h post-IR, Fibroblasts displayed decreased contacts between chromosome arms. GM12878, however, had a variable response, with an overall initial gain in interactions that were lost by 24 h (Sanders et al., 2020). These differing responses could be due to cell cycle phase, repair kinetics, or potential differences in the type of breaks such as single-stranded to double-stranded break ratio. IR failed to induce consistent changes at the A/B compartment level, but did promote significant increases in TAD boundary strength that remained up to 5 days post IR in all cell types tested (Sanders et al., 2020). Like nuclease-induced breaks, the increase in TAD insulation was dependent on ATM (Sanders et al., 2020). Additionally, cohesin binding was reinforced after IR, (Kim et al., 2010), which may contribute to the increased TAD boundary strength. Why TADs are more insulated following IR remains an open question.

## Discussion

In this review, we have highlighted the role for the 3D genome in the processes of DNA replication, DNA repair and cell cycle progression. While extensive progress has been made connecting genome structure and function, our understanding is far from complete. The development of degron models, such as dTAG, Auxin-degron (AID) and PROTAC, to rapidly deplete chromatin architectural proteins can help further resolve the cell cycle kinetics of chromatin architecture in different cell types and disease models. For example, depletion of cohesin with AID has demonstrated rapid loss of chromatin loops following, of which the majority were reformed within 1 h after releasing from AID-directed protein degradation (Rao et al., 2017). Collectively degron studies degrading chromatin architectural proteins, including cohesin, CTCF and WAPL, to study gene regulation have identified that while local enhancer-promoter interactions are lost, there is only a modest effect on transcription and no significant change to histone modifications or A/B compartments (Hsieh et al., 2022; Hyle et al., 2023; Kriz et al., 2021; Luan et al., 2021; Rao et al., 2017). To date, these studies have not considered the implications for DNA replication, repair or cell cycle kinetics.

Higher-resolution 3D genome maps such as through Micro-C (Hamley et al., 2023) and Hi-ChIP (Bhattacharyya et al., 2019) can also help gain a greater understanding of how these regulating factors control chromatin loops. Leveraging these advanced tools will be pivotal in uncovering the intricacies of chromatin dynamics at higher resolution in a time resolved manner. Unifying our understanding of DNA replication at different genomic scales will also be critical to understanding how thousands of replication forks are deployed to copy genetic and epigenetic information. Advances in BrdU and EdU detection using nanopore sequencing may supersede current DNA fibre assays and provide a much needed technique to bridge our knowledge of replication forks dynamics with genome organisation and epigenetic regulation (Hennion et al., 2020; Jones et al., 2022; Muller et al., 2019).

Understanding 3D genome plasticity provides critical insights into the mechanisms underlying various diseases and their treatments, as there is evidence TAD structure is dynamically altered within diseases such as cancer and neurodegeneration (Dileep et al., 2023; Taberlay et al., 2016). In cancer, the 3D genome can be remodelled upon exposure to epigenetic or chemotherapy, leading to decreased cancer associated gene expression and reduced proliferation (Achinger-Kawecka et al., 2024; Achinger-Kawecka et al., 2020). Cohesin subunit STAG2 has been identified as synthetic lethal with STAG1, suggesting chromatin architectural proteins may be a promising therapeutic target (van der Lelij et al., 2020). Further, new druggable regulators of the 3D genome have been discovered through a high throughput screen (Park et al., 2023), highlighting how the 3D genome can be targeted for improved patient outcomes. Understanding the intricate dynamics of the 3D genome and genome stability not only holds promise for refining therapeutic approaches but also offers invaluable insights into a myriad of genomic technologies, paving the way for transformative advancements in both healthcare and research.
